# Effects of Dose and Duration of Zinc Interventions on Risk Factors for Type 2 Diabetes and Cardiovascular Disease: A Systematic Review and Meta-Analysis

**DOI:** 10.1093/advances/nmaa087

**Published:** 2020-07-28

**Authors:** Laura M Pompano, Erick Boy

**Affiliations:** HarvestPlus, International Food Policy Research Institute, Washington, DC, USA; HarvestPlus, International Food Policy Research Institute, Washington, DC, USA

**Keywords:** zinc biofortification, fortification, diabetes mellitus, cardiovascular disease, zinc supplementation, noncommunicable disease, chronic disease, zinc interventions

## Abstract

No meta-analysis has examined the effect of dose and duration of zinc interventions on their impact on risk factors for type 2 diabetes (T2D) or cardiovascular disease (CVD). This study aimed first to compare the effects of zinc interventions dichotomized as low versus high dose (<25 mg/d and ≥25 mg/d, respectively) and short versus long duration (<12 wk and ≥12 wk, respectively) on risk factors for T2D and CVD. Second, it discusses the results from the low-dose and long-duration meta-analyses as a foundation for understanding what impact a zinc-biofortification intervention could have on these risk factors. The PubMed and Cochrane Review databases were searched through January 2020 for full-text, human studies providing zinc supplements (alone) at doses ≤75 mg/d and a placebo. Data on study and sample characteristics and several T2D and CVD risk factors were extracted. There were 1042 and 974 participants receiving zinc and placebo, respectively, from 27 studies. Low-dose zinc supplementation (<25 mg/d) significantly benefited fasting blood glucose, insulin resistance, triglycerides, total cholesterol, and LDL cholesterol. High-dose zinc supplementation (≥25 mg/d) benefited glycated hemoglobin and insulin resistance. Short-duration interventions (<12 wk) benefited fasting blood glucose, insulin resistance, and triglycerides, while long-duration studies (≥12 wk) benefited fasting blood glucose, triglycerides, and total and LDL cholesterol. Effect sizes for low-dose and long-duration interventions were of equal or greater magnitude to those from high-dose or short-duration interventions. Low-dose and long-duration zinc supplementation each improved more risk factors for T2D and CVD than high-dose and short-duration interventions, respectively. It is currently unknown whether low doses of zinc delivered over long durations via a biofortified crop would similarly impact these risk factors. However, this review suggests that low-dose, long-duration zinc intake from supplements, and potentially biofortification, can benefit risk factors for T2D and CVD.

## Introduction

Noncommunicable diseases (NCDs) such as type 2 diabetes (T2D) and cardiovascular disease (CVD) account for more deaths globally than any other condition (1). In 2018, the WHO reported that NCDs accounted for 71% of global deaths (2). They also showed that low- and middle-income countries are disproportionately affected by NCDs, accounting for 85% of NCD-related deaths among individuals aged 30–69 y (2). Among NCDs, CVD and T2D are the leading and fourth-leading causes of death, respectively, collectively accounting for 19.5 million deaths worldwide in 2018. Furthermore, despite increasing global awareness, the prevalence of these conditions continues to increase at alarming rates. For example, the global prevalence of T2D is expected to reach 13.9% by 2030 from a prevalence of 9.1% in 2014 (3), while deaths from CVD are expected to reach 23.6 million annually by 2030 from 17.6 million deaths in 2016 (4). The underlying etiology of these conditions is complex, as they can be influenced by a number of environmental, genetic, and behavioral factors (5, 6). However, diet and nutrition play a particularly important role in these conditions, especially in the context of the double burden of malnutrition facing many low- and middle-income countries.

The double burden of malnutrition is the existence of both over- and undernutrition within a population, household, or individual. This can manifest as the simultaneous presence of NCDs, overweight, or obesity alongside ≥1 micronutrient deficiencies within an individual or household. However, it can also be experienced over a lifetime or across generations in the form of early-childhood malnutrition and stunting, which can predispose individuals towards obesity or NCDs later in life (7). Zinc is especially relevant in this context, as its deficiency is known to impair immune competence (8), proper growth and development in children (9), and is related to the pathophysiology of T2D and CVD in adults (10–13). The relations between zinc and the pathophysiology of T2D and CVD have been summarized extensively (14–16); however, a brief summary of relevant literature is provided here.

Zinc affects multiple aspects of insulin homeostasis and the inflammatory response in T2D. It is critical for proper secretion of insulin from pancreatic β cells (15), contributes to insulin transport and binding to its receptor on other cells (17), and can impact insulin sensitivity and resistance by activating multiple cell signaling cascades (10, 18). Zinc-deficient β cells have been shown to have fewer insulin granules and suffer from greater oxidative stress than zinc-replete cells (14), effects that are primarily a result of changes in metal-binding metallothionein proteins as well as zinc transporters of the Zrt- and Irt-like protein (ZIP) and zinc transporter (ZnT) families, among which, ZnT8 has been shown to be particularly important for the pathophysiology of T2D (14, 19).

Zinc also plays a role in lipid metabolism through antioxidant, anti-inflammatory, and other mechanisms that can alter atherosclerosis and the risk of CVD (16, 20). Atherosclerosis is typically associated with increased oxidative stress, which damages endothelial cells, alters inflammatory signaling, and can modify low density lipoprotein (LDL) in ways that promote atherogenesis (16). One key mechanism for dealing with oxidative stress is the use of antioxidant enzymes including Cu-Zn superoxide dismutase, catalase, and others (20–22). While zinc is not redox reactive itself, it is a cofactor for Cu-Zn superoxide dismutase and is involved in the regulation of several other antioxidant enzymes (23). It is also critical for proper clearing of reactive oxygen and nitrogen species (16). As such, zinc deficiency exacerbates oxidative stress, and ultimately CVD, by increasing production of reactive oxygen species, promoting apoptosis of endothelial and vascular smooth muscle cells, activating proinflammatory cytokines, and amplifying the oxidation of LDL (24–26).

There is conflicting literature on whether zinc supplementation impacts blood pressure, with some studies reporting an inverse relation between zinc and hypertension (27, 28) while others report a direct relation between the 2 (29, 30). While some mechanisms have been proposed relating zinc to hypertension, including zinc's role in ATP-dependent calcium pumps (31) as well as in the NO pathway (32), the relation between zinc and blood pressure is currently unclear.

Despite these critical roles in health, zinc deficiency remains a large global health concern, with 17.3% of the world's population facing insufficient zinc intake, a rate which may grossly underestimate the true burden of zinc deficiency as it relates to stunting, diarrhea, pneumonia, and other conditions (33, 34).

Common strategies for addressing zinc deficiency include industrial food fortification, multiple micronutrient powders or supplements, increasing dietary diversity, and zinc supplementation (35). Collectively, these methods can address the nutritional needs of a large portion of the population. However, low-resource and rural communities often cannot afford to purchase a diverse diet, commercially fortified products, or zinc supplements. As a result, they are often left unprotected, despite being among those with the greatest risk of zinc deficiency (35).

To help breach this gap, biofortification of staple crops with essential micronutrients has been introduced in several countries. Biofortification is the use of conventional plant breeding and agronomic practices to increase the nutrient density of staple crops (36). To date, only a few studies have examined the efficacy of zinc biofortification in improving human health. One of these studies found that consumption of zinc-biofortified wheat reduced pneumonia and vomiting in children and significantly reduced the number of days with fever in women of child-bearing age in Delhi, India (37). In contrast, several studies of crops biofortified with iron or provitamin A carotenoids have demonstrated the efficacy of using biofortification to improve the total nutrient intake and status of the populations consuming them (38–40).

Biofortification provides a lower nutrient dose than other intervention strategies. However, this dose can be maintained indefinitely with little to no input after the adoption of the improved varieties due to the self-sustaining nature of the intervention (farmers saving and sharing seeds, etc.). As a result, biofortified crops could sustainably add low doses of zinc to the diet over all stages of the life course. Currently, the impacts of a long-duration, low-dose zinc intervention remain unclear both in terms of how such an intervention could impact zinc deficiency and how it would affect conditions like T2D and CVD, which are aggravated by, but not caused by, zinc deficiency (13).

Several recent meta-analyses have examined the effects of zinc supplementation on risk factors for T2D and CVD (10, 41–48). Their results have been somewhat mixed, with some recent studies finding that very high intakes of zinc were associated with increased risk for chronic disease (49, 50), while others reported that zinc supplementation benefited risk factors for T2D and CVD including glycemic control and lipid metabolism (10, 16, 42, 46). Interestingly, many studies reporting a negative effect of zinc on NCD risk found this effect as zinc dosage was increased. However, to date, no meta-analysis has examined the influence of the dose or duration of zinc supplementation provided on risk factors for T2D and CVD. Additionally, despite the increase in its use as a zinc intervention, to our knowledge, no study has ever examined the effects of consuming zinc-biofortified crops on the risk of T2D or CVD.

In light of these gaps in the literature, this study aimed to compare the effects of low- versus high-dose as well as short- versus long-duration zinc interventions on risk factors for T2D [fasting blood glucose (FBG), glycated hemoglobin (HbA1c), and the HOMA-IR] and CVD [triglycerides (TGs), total cholesterol (TC), LDL cholesterol, HDL cholesterol, systolic blood pressure (SBP), and diastolic blood pressure (DBP)]. We then discuss the results from the low-dose and long-duration zinc supplementation meta-analyses as a foundation for understanding what impact a zinc-biofortification intervention could have on these risk factors.

## Methods

### Information sources and literature search

Studies were identified by searching PubMed and the Cochrane database and by scanning reference lists of reviews and articles. The literature search was conducted for studies published before 31 January 2020 (last search date).

The search terms for T2D were as follows: “Zinc” [Title/Abstract] AND (“Diabetes Mellitus” OR “Glucose Intolerance” OR “Intolerance, Glucose” OR “Impaired Glucose Tolerance” OR “Glucose Tolerance, Impaired” OR “Tolerance, Impaired Glucose” OR “Tolerances, Impaired Glucose” OR “diabetes” OR “HbA1c” OR “glycated hemoglobin” OR “HOMA-IR” OR “insulin sensit*” OR “metabolic disease” OR “metabolic syndrome”) [all fields]. The search terms for CVD were “Zinc” [Title/Abstract] AND “(Cardiovascular” OR “cardiovascular” OR “heart disease” OR “coronary” OR “Hypertension” OR “hypertensi*” OR “hyperlipid*” OR “hypercholesterol*” OR “hyperlipoprotein*” OR “hypertriglycerid*” OR “Arteriosclero*” OR “cholesterol” OR “blood pressure”) [all fields].

### Inclusion and exclusion criteria

Study inclusion and exclusion criteria are detailed in the PICOS (Population Intervention Comparison Outputs) table in **Supplemental Table 1**. Only full-text, human studies were included in the analysis. No publication date restrictions were imposed. For studies producing multiple publications, only the first publication was included. If later publications contained other outcomes of interest, data from the later publication were included only for the additional outcome(s). Studies providing pharmaceutical doses of zinc (defined as ≥100 mg/d) were excluded from the present analysis, which was only interested in the effects of supplemental zinc as typically administered in zinc interventions.

### Data extraction

The following data were extracted from all included studies by 1 author (LMP): name of the first author, year of publication, publishing journal, sample size, participant sex and age at baseline, health status of participants (obese, healthy, T2D, etc.), intervention dose (in milligrams per day) and duration (in weeks), and data for any included outcomes of interest (mean and SD).

### Risk of bias

Risk of bias was assessed using the Cochrane Collaboration risk-of-bias tool in RevMan 5.3 statistical software (Review Manager, Copenhagen: The Nordic Cochrane Centre, The Cochrane Collaboration, 2011) (51). All domains were included in the risk-of-bias table (random-sequence generation, allocation concealment, blinding of participants and personnel, blinding of outcome assessment, incomplete outcome data, selective reporting, and other bias). Studies were defined as high risk if they had ≥2 high-risk domains.

Publication bias was assessed using funnel plots for analyses with ≥10 studies (FBG, HbA1c, HOMA-IR, TGs, TC, LDL cholesterol, and HDL cholesterol). Publication bias was not assessed for analyses with ≤9 included studies as the power would be too low to distinguish true bias from chance (SBP and DBP) (51).

### Statistical analysis

Pairwise meta-analyses of the included studies were conducted for each outcome of interest using RevMan 5.3. For each analysis, the pooled mean difference was calculated between intervention groups. Statistical heterogeneity between studies was assessed using a cutoff of *I*^2^ >50% to define substantial heterogeneity (52). Random-effects meta-analyses were used for all comparisons due to high heterogeneity in the fixed-effects models, based on the *I*^2^ cutoff. Statistical significance was defined as a *P* value <0.05.

Two studies [Black et al. (53) and Hininger-Favier et al. (54)] had 3 eligible treatment arms (placebo and 2 doses of zinc). All analyses were run as follows: including only dose 1 versus placebo, including dose 2 versus placebo, and including both dose 1 versus placebo and dose 2 versus placebo. All combinations of the 4 possible arms were examined when Black et al. (53) and Hininger-Favier et al. (54) were in the same analysis. No differences in magnitude or direction of the meta-analyses results were observed for any combination of these sets of comparisons (data not shown). Therefore, all 4 comparisons were included in the meta-analyses. Black et al. (53) had data for TC and HDL cholesterol (high-dose, long-duration). Hininger-Favier et al. (54) had data for TGs, TC, LDL cholesterol, and HDL cholesterol (high-dose, low-dose, and long-duration).

Boukaiba et al. (55), Crouse et al. (56), and Oh et al. (57) each had 2 independent sets of data (zinc vs placebo in low and normal BMI individuals, sedentary and aerobically trained individuals, and patients with and without T2D, respectively). As there were no overlapping data for these 6 datasets, all were retained in the present analyses.

Finally, 2 crossover studies [Hashemipour et al. (58) and Parham et al. (59)] presented each wave of data separately rather than showing combined data. Each wave was included as a separate dataset. After the addition of the 2 datasets from Black et al. (53) and Hininger-Favier et al. (54), the 6 independent datasets in Boukaiba et al. (55), Crouse et al. (56), and Oh et al. (57), and the 2 crossover studies, there were 34 datasets in the present analyses from the 27 included publications.

TGs, TC, LDL cholesterol, and HDL cholesterol were reported as milligrams per deciliter. Data from studies reporting TC, LDL cholesterol, or HDL cholesterol in millimoles per liter were converted to milligrams per deciliter by multiplying the millimole/liter value by 38.67. TG values reported in millimoles per liter were converted by multiplying the millimole/liter value by 88.57 (60). Zinc doses are reported as doses of elemental zinc (milligrams per day), not the dose of the compound in which it was delivered.

To best reflect the doses of zinc that are feasible through biofortification, a low dose was defined as ≤25 mg elemental Zn/d, while high doses were defined as 25–75 mg/d. Duration was defined as short or long for studies that provided zinc supplementation for <12 wk or ≥12 wk, respectively.

The combined analyses for each of the 9 outcomes were also done excluding studies that had 2 high-risk domains in the risk-of-bias assessment to understand how they influenced the results.

### Ethics approval

No ethical approval was required as only data from previous studies that had already obtained informed consent were retrieved and analyzed.

## Results

### Literature search

The literature search was conducted according to the search criteria specified. The T2D and CVD searches returned 1474 studies and 1993 studies, respectively. The titles of these publications were screened to remove duplicates and eliminate irrelevant articles. Next, we conducted an abstract screening of 194 studies and 86 literature reviews that were selected from the title screening. Of these, 23 articles and 72 literature reviews were selected for full-text review. The full-text screening and manual searches of reference lists identified 27 individual publications that met the inclusion criteria for the present analysis (53–59, 61–80). As discussed in the Methods, these 27 publications contained a total of eligible 34 datasets that were included in the present analyses. The study selection and review were conducted by 1 author (LMP); questions about eligibility of articles or reviews were deliberated with a second member of the research group until a consensus was reached. The characteristics of the studies included in the present analyses are presented in **Table 1**.

**TABLE 1:** Characteristics of included randomized controlled studies comparing the effects of supplemental zinc or placebo on risk factors for T2D or cardiovascular disease^1^

Study (ref)	Participants and design, *n*	Dose, mg elemental Zn/d	Duration of zinc supplementation	Sex	Age, y	Population health status	Outcomes measured
Al-Maroof et al. (78)	Zn, 43 Placebo, 43	30	3 mo	Both	54.6 ± 9.2	T2D	FBG, HbA1c
Black et al. (53)	Zn (dose 1), 13 Zn (dose 2), 9 Placebo, 9	50 75	12 wk	Males	19 to 29	Healthy	TGs, TC, LDL-C, HDL-C
Boukaiba et al. (55)	Crossover, 44	20	8 wk	Both	87 ± 1.0	Healthy	TGs, TC, LDL-C, HDL-C
Crouse et al. (56)	Zn, 23 Placebo, 21	28.7	8 wk	Men	20 to 55	Healthy	TGs, TC, LDL-C, HDL-C
El-Ashmony et al. (79)	Zn, 26 Placebo, 30	9.2	8 wk	Both	30 to 70	T2D	FBG, HbA1c, TGs, TC, LDL-C, HDL-C
Foroozanfard et al. (80)	Zn, 26 Placebo, 26	50	8 wk	Females	24 to 26	PCOS	FBG, HOMA-IR, TGs, TC, LDL-C, HDL-C
Foster et al. (61)	Zn, 12 Placebo, 10	40	12 wk	Females	65 ± 7.8	T2D	HbA1c, TGs, TC, LDL-C, HDL-C
Gatto and Samman (62)	Crossover, 10	50	4 wk	Males	24.3 ± 4.2	Healthy	TGs, TC, LDL-C, HDL-C
Gómez-García et al. (63)	Zn, 7 Placebo, 7	23	30 d	Males	21 to 30	T2D	TGs, TC, LDL-C, HDL-C
Hashemipour et al. (58)	Crossover, 60	20	8 wk	Both	6 to 10	Healthy (obese)	FBG, HOMA-IR, TGs, TC, LDL-C, HDL-C, SBP, DBP
Hininger-Favier et al. (54)	Zn (dose 1), 126 Zn (dose 2), 131 Placebo, 130	15 30	6 mo	Both	55 to 85	Healthy	TC, LDL-C, HDL-C
Islam et al. (64)	Zn, 28 Control, 27	30	6 mo	Both	30 to 65	Prediabetes	FBG, TGs, LDL-C, HDL-C
Karamali et al. (65)	Zn, 29 Placebo, 29	30	6 wk	Females	18 to 40	Gestational diabetes	FBG, HOMA-IR, TGs, TC, LDL-C, HDL-C
Khan et al. (66)	Zn, 23 Usual care, 21	50	12 wk	Both	40 to 69	T2D	FBG, TGs, TC, LDL-C, HDL-C
Kim and Lee (67)	Zn, 20 Placebo, 20	30	8 wk	Females	18 to 28	Healthy (obese)	FBG, HOMA-IR, TGs, TC, HDL-C, SBP, DBP
Marreiro et al. (68)	Zn, 28 Placebo, 28	30	30 d	Females	24 to 45	Healthy (obese)	FBG, HOMA-IR
Momen-Heravi et al. (69)	Zn, 30 Placebo, 28	50	12 wk	Both	40 to 85	T2D	FBG, HbA1c, HOMA-IR, TGs, TC, LDL-C, HDL-C
Nazem et al. (70)	Zn, 35 Placebo, 35	50	8 wk	Both	40 to 65	T2D	FBG, HbA1c, HOMA-IR, TC, LDL-C, HDL-C
Oh and Yoon (57)	T2D: Zn, 44 Placebo, 32 Healthy: Zn, 32 Placebo, 40	50 50	4 wk	Both	1. 49.6 ± 10.7 2. 58.7 ± 10.1	1. T2D 2. Healthy	FBG, HBA1c
Parham et al. (59)	Crossover, 39	30	3 mo	Both	52.0 ± 9.3 to 54.5 ± 9.2	T2D	FBG, HbA1c, TGs, TC, LDL-C, HDL-C, SBP, DBP
Partida-Hernández et al. (71)	Crossover, 27	23	12 wk	Males	35 to 65	T2D	FBG, HbA1c, TGs, TC, LDL-C, HDL-C
Payahoo et al. (72)	Zn, 30 Placebo, 30	30	4 wk	Both	18 to 45	Healthy (obese)	FBG, TGs, TC, LDL-C, HDL-C
Payahoo et al. (73)	Zn, 30 Placebo, 30	30	4 wk	Both	18 to 45	Healthy (obese)	HOMA-IR
Rahimi-Ardabili et al. (74)	Zn, 30 Placebo, 30	23	60 d	Both	52.8 ± 12.7	HD patients	TGs, TC, LDL-C, HDL-C
Ranasinghe et al. (81)	Zn, 100 Placebo, 100	20	12 mo	Both	51.8 ± 7.3	Prediabetic	FBG, HOMA-IR, TC, LDL-C
Roozbeh et al. (76)	Zn, 27 Control, 26	50	6 wk	Both	55.7 (no SD reported)	HD patients	TGs, TC, LDL-C, HDL-C
Roshanravan et al. (77)	Zn, 22 Placebo, 22	30	8 wk	Females	29.5 ± 4.2 to 29.8 ± 5.4	Pregnant, impaired glucose tolerance	FBG, HOMA-IR

^1^DBP, diastolic blood pressure; FBG, fasting blood glucose; HbA1c, glycated hemoglobin; HD, hemodialysis; HDL-C, HDL cholesterol; LDL-C, LDL cholesterol; PCOS, polycystic ovarian syndrome; ref, reference; SBP, systolic blood pressure; TC, total cholesterol; TG, triglyceride; T2D, type 2 diabetes.

### Description of included studies

**FIGURE 1:**
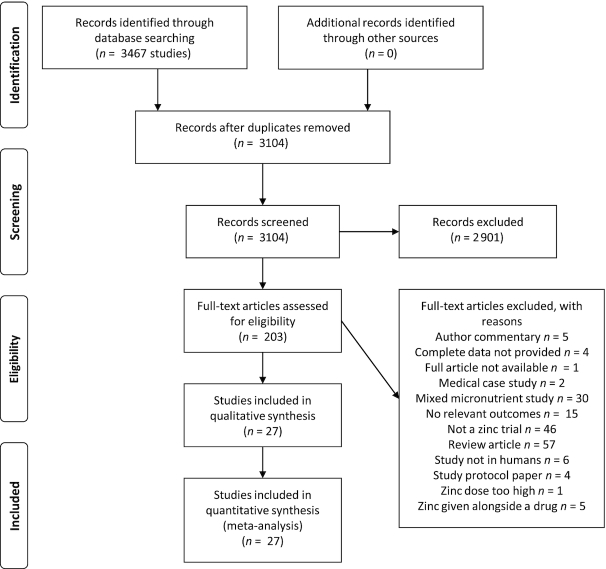
PRISMA flow chart for study selection. PRISMA, Preferred Reporting Items for Systematic Reviews and Meta-Analyses.

A flowchart of study selection and inclusion is shown in **Figure 1**. All studies that were included in the meta-analyses were human trials that compared zinc supplementation with a placebo or control. The duration of zinc supplementation ranged from 4 wk to 12 mo, with mean and median durations of 11.0 wk and 8 wk, respectively. Elemental zinc supplementation doses ranged from 9.8 mg/d to 75 mg/d. The mean and median doses of elemental zinc across studies were 34.4 mg/d and 30.0 mg/d, respectively. Zinc was delivered as zinc sulfate (*n* = 13 studies), zinc gluconate (*n* = 10), and zinc amino chelate (*n* = 1), and 3 studies did not identify the form of zinc provided.

A total of 1042 participants were assigned to receive zinc supplementation while 974 received control, usual care, or a placebo. Participant age spanned from 6 to 106 y, with the majority of studies including adults between the ages of 20 and 70 y. Of the included studies, 9 studies involved T2D patients, 5 studies included healthy normal-weight patients, 6 studies included healthy but obese patients, 1 study involved lean healthy patients, 2 studies involved hemodialysis patients, 2 studies involved patients with prediabetes, 1 study involved patients with polycystic ovarian syndrome, 1 study had patients with gestational diabetes, and 1 study had participants who were pregnant and had impaired glucose tolerance.

**FIGURE 2:**
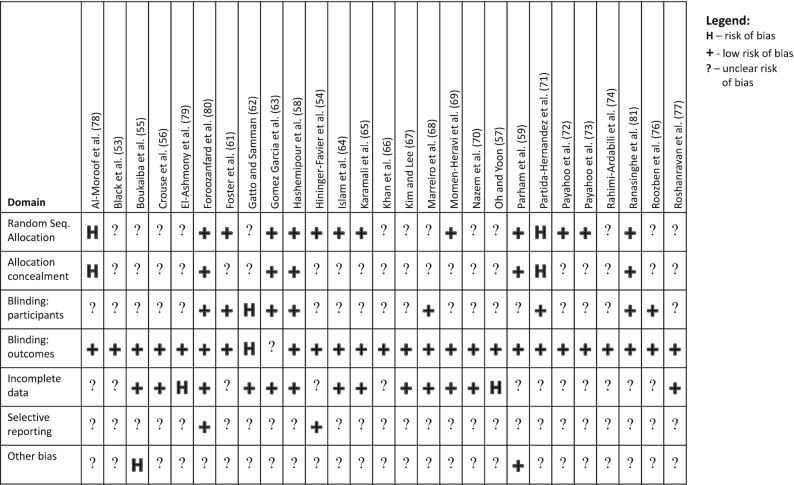
Risk-of-bias assessment by study and domain. Bias rankings were based on the Cochrane Review Risk of Bias tool and guidelines (51). Allocation concealment, allocation concealment (selection bias); Blinding: outcomes, blinding of outcome assessment (detection bias); Blinding: participants, blinding of participants and personnel (performance bias); Incomplete data, incomplete outcome data (attrition bias); Random Seq. Allocation, random-sequence allocation (selection bias); Selective reporting, selective reporting (reporting bias).

Risk of bias in individual studies was assessed using the risk-of-bias tool in Review Manager version 5.3. Out of the 27 included studies, 4 had a high risk of bias in 1 domain and 3 studies had high risk in 2 domains (**Figure 2**). Excluding studies with 2 high-risk domains (*n* = 3 studies excluded) did not meaningfully alter the significance, direction, or size of the effect for any outcome in the duration analyses (all risk factors, long or short duration) or the majority of the dose analyses (TGs, TC, LDL cholesterol, or HDL cholesterol low or high dose, and FBG and HbA1c low dose)(data not shown). Excluding these analyses did change the significance level but not the magnitude or direction of effect for high-dose FBG [effect size (ES) including all datasets: −6.68; 95% CI: −13.62, 0.27; ES when high-risk studies were excluded (*n* = 2 excluded): −7.15; 95% CI: −14.20, −0.14] and high-dose HbA1c [ES including all datasets: −0.37; 95% CI: −0.71, −0.03; ES when high-risk studies were excluded (*n* = 2 excluded): −0.37; 95% CI: −0.79, 0.04].

Publication bias was assessed using funnel plots. **Supplemental Figure 1** shows the funnel plots of the mean differences for analyses with >10 studies, the minimum number of studies suggested for use of the tool (51). Visual analysis of the funnel plots suggested that FBG, TGs, TC, and LDL cholesterol were symmetric and thus at low risk for publication bias. The funnel plot for HDL cholesterol was mostly symmetric but had 2 analyses (low-dose and long-duration) that each had 1 study noticeably different than the others, which may indicate asymmetry and publication bias, or may be a result of only having 10 and 12 studies included in each analysis, respectively.

### Effects by dose

Random-effects meta-analyses were used in all analyses due to the high heterogeneity (*I*^2^ >50%). Seven publications provided a low dose of zinc (<25 mg/d) and 18 provided a high dose of zinc (≥25 mg/d). Hininger-Favier et al (54) had 2 zinc treatment arms, 1 providing a low dose (15 mg/d) and another providing a high dose (30 mg/d). Black et al. (53) also provided 2 doses, both of which were high dose (50 mg/d and 75 mg/d). In total, 8 studies provided low-dose supplementation and 19 provided high-dose supplementation. The Pearson correlation between dose (in milligrams per day) and duration (in months) was *r* = −0.27 (*P* = 0.18). Because there was no association between dose and duration, their effects were examined separately.

**FIGURE 3:**
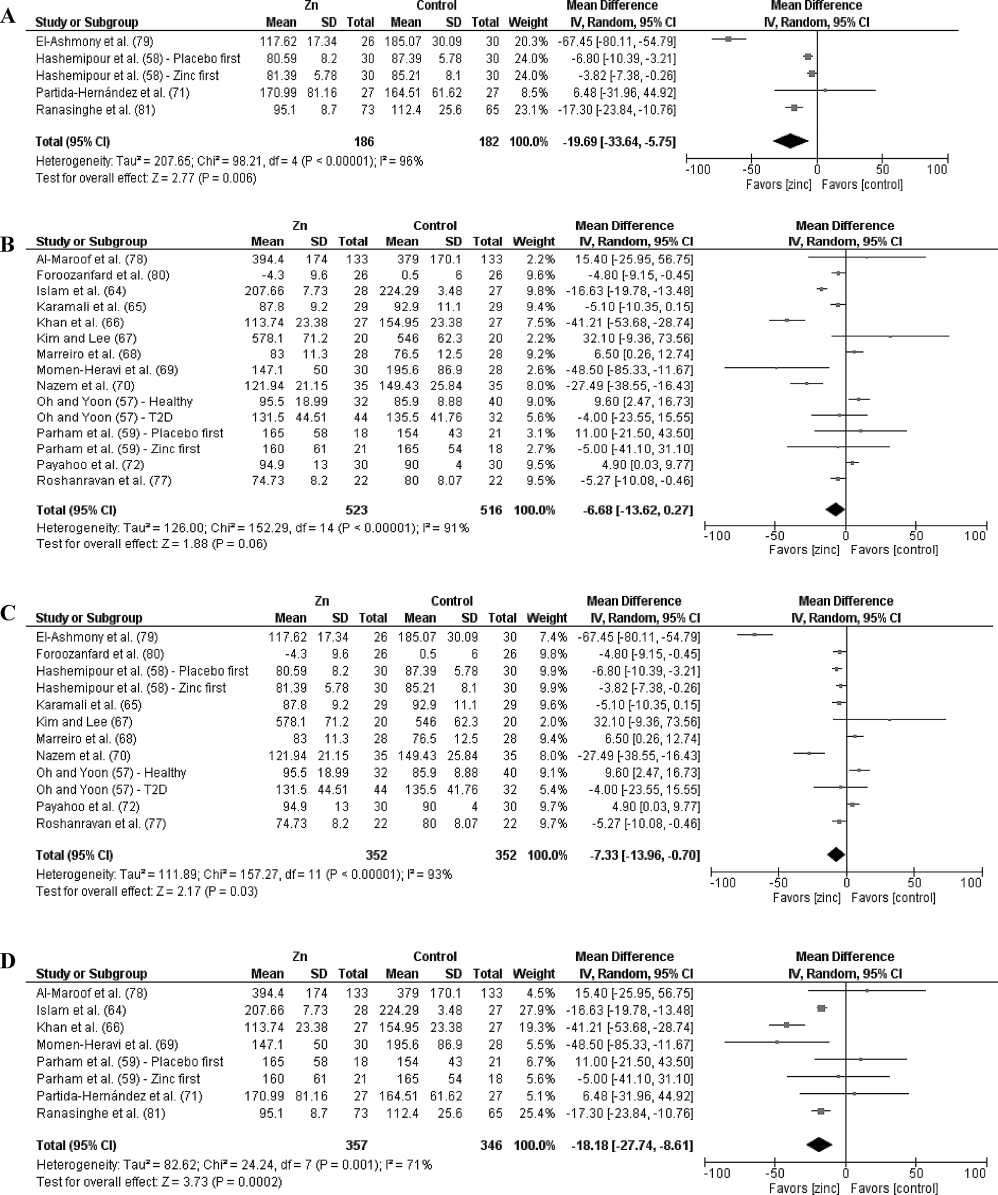
Meta-analyses of the mean difference between zinc and placebo for fasting blood glucose, by dose and duration. (A) Studies providing low-dose supplementation, defined as <25 mg elemental Zn/d. (B) Studies providing high-dose supplementation, defined as ≥25 mg elemental Zn/d. (C) Studies providing short-duration supplementation, defined as <12 wk. (D) Studies providing long-duration supplementation, defined as ≥12 wk. IV, inverse variance.

**FIGURE 4:**
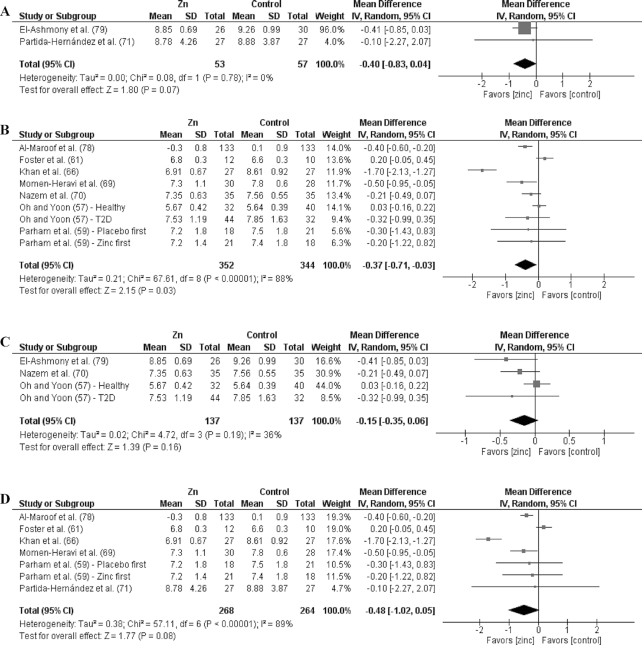
Meta-analyses of the mean difference between zinc and placebo for HbA1c, by dose and duration. (A) Studies providing low-dose supplementation, defined as <25 mg elemental Zn/d. (B) Studies providing high-dose supplementation, defined as ≥25 mg elemental Zn/d. (C) Studies providing short-duration supplementation, defined as <12 wk. (D) Studies providing long-duration supplementation, defined as ≥12 wk. HbA1c, glycated hemoglobin; IV, inverse variance.

**FIGURE 5:**
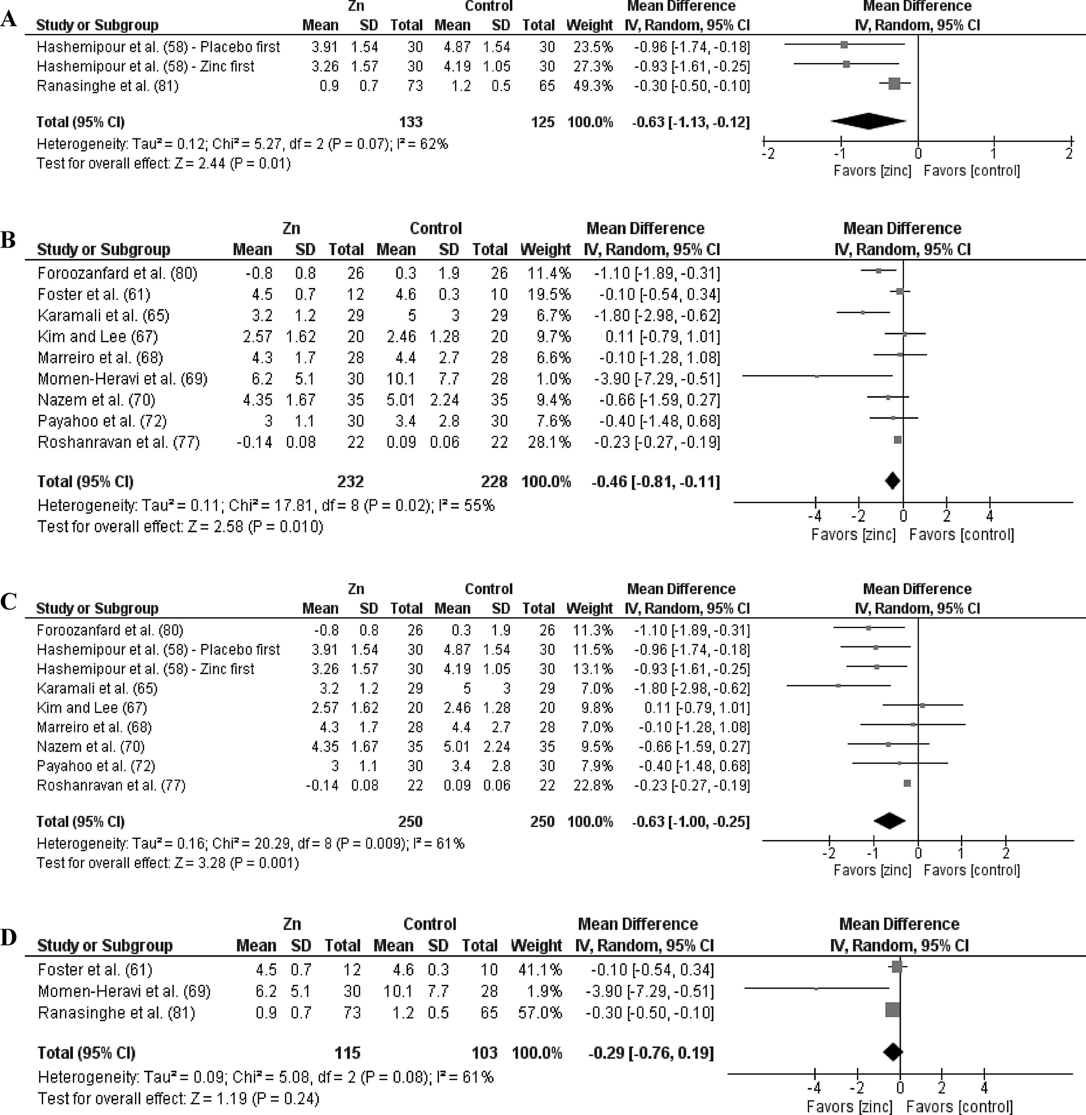
Meta-analyses of the mean difference between zinc and placebo for HOMA-IR, by dose and duration. (A) Studies providing low-dose supplementation, defined as <25 mg elemental Zn/d. (B) Studies providing high-dose supplementation, defined as ≥25 mg elemental Zn/d. (C) Studies providing short-duration supplementation, defined as <12 wk. (D) Studies providing long-duration supplementation, defined as ≥12 wk. IV, inverse variance.

**FIGURE 6:**
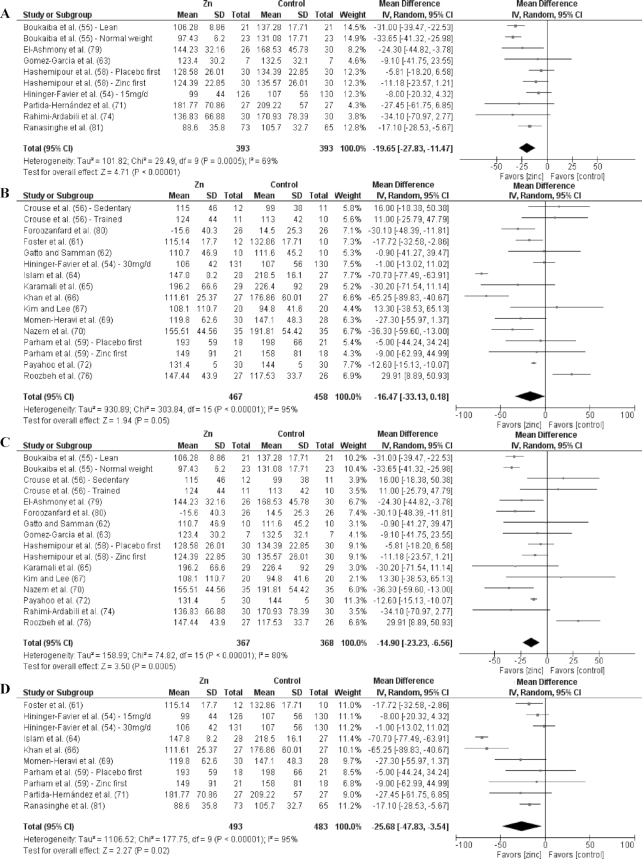
Meta-analyses of the mean difference between zinc and placebo for triglyceride concentration, by dose and duration. (A) Studies providing low-dose supplementation, defined as <25 mg elemental Zn/d. (B) Studies providing high-dose supplementation, defined as ≥25 mg elemental Zn/d. (C) Studies providing short-duration supplementation, defined as <12 wk. (D) Studies providing long-duration supplementation, defined as ≥12 wk. IV, inverse variance.

**FIGURE 7:**
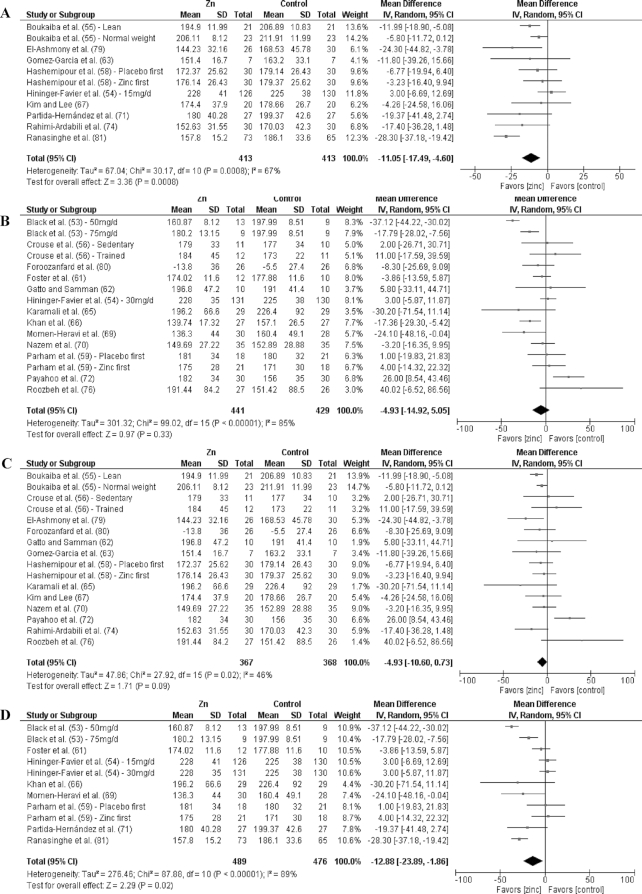
Meta-analyses of the mean difference between zinc and placebo for total cholesterol, by dose and duration.(A) Studies providing low-dose supplementation, defined as <25 mg elemental Zn/d. (B) Studies providing high-dose supplementation, defined as ≥25 mg elemental Zn/d. (C) Studies providing short-duration supplementation, defined as <12 wk. (D) Studies providing long-duration supplementation, defined as ≥12 wk. IV, inverse variance.

**FIGURE 8:**
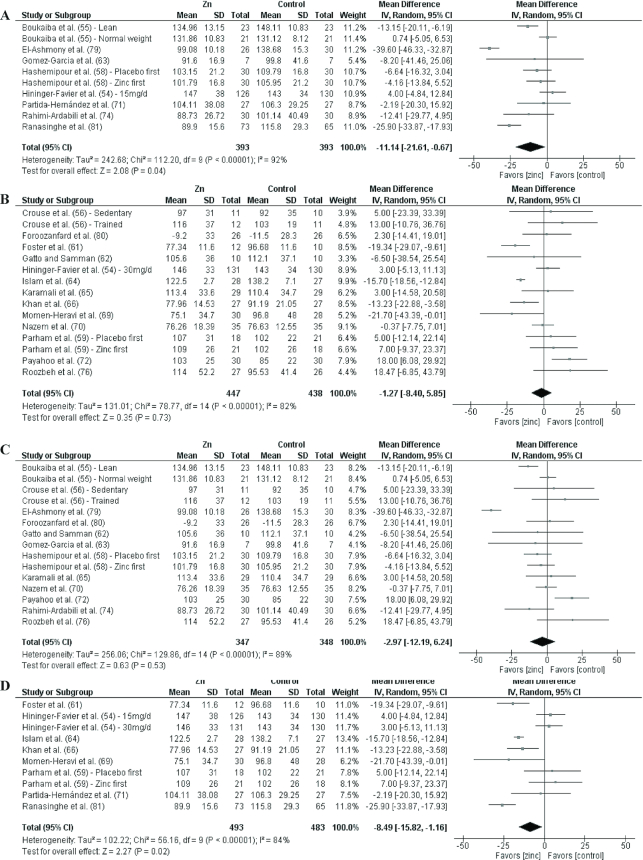
Meta-analyses of the mean difference between zinc and placebo for LDL cholesterol, by dose and duration. (A) Studies providing low-dose supplementation, defined as <25 mg elemental Zn/d. (B) Studies providing high-dose supplementation, defined as ≥25 mg elemental Zn/d. (C) Studies providing short-duration supplementation, defined as <12 wk. (D) Studies providing long-duration supplementation, defined as ≥12 wk. IV, inverse variance.

**FIGURE 9:**
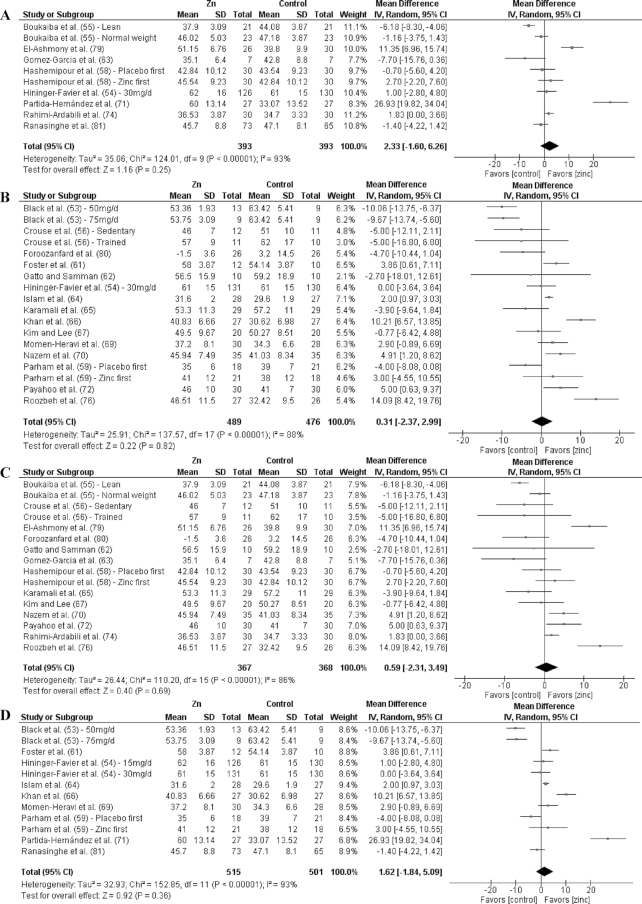
Meta-analyses of the mean difference between zinc and placebo for HDL cholesterol, by dose and duration. (A) Studies providing low-dose supplementation, defined as <25 mg elemental Zn/d. (B) Studies providing high-dose supplementation, defined as ≥25 mg elemental Zn/d. (C) Studies providing short-duration supplementation, defined as <12 wk. (D) Studies providing long-duration supplementation, defined as ≥12 wk. IV, inverse variance.

Analyses by dose are shown in **Figures 3**–**9** as well as in **Supplemental Figures 2** and **3**. For T2D outcomes, FBG and HOMA-IR showed significant improvements from low-dose zinc supplementation compared with placebo while HbA1c and HOMA-IR improved from high-dose supplementation compared with placebo. The ES for the low-dose supplementation was greater than that of the high-dose for both FBG and HOMA-IR.

The effect of dose on the CVD risk factors was similar, with low-dose zinc supplementation showing significant, beneficial effects for TGs, TC, and LDL cholesterol. In contrast, high-dose supplementation only showed significant effects for TGs. The ESs for the low-dose supplementation studies were greater than the ESs for the high-dose studies for TGs, TC, and LDL cholesterol. There was no effect of zinc supplementation on HDL cholesterol for either low- or high-dose supplementation.

No effects were observed on SBP or DBP for low- or high-dose supplementation (Supplemental Figures 2 and 3).

### Effects by duration

Of the 27 studies, 17 were short duration and 10 were long duration. Analyses by duration are shown in Figures 3–9 as well as in Supplemental Figures 2 and 3. Among the T2D risk factors, short-duration supplementation showed significant benefits for FBG and HOMA-IR. Long-duration supplementation showed significant benefits for FBP and HbA1c. The ES of long-duration studies on FBG was more than double the effect of short-duration studies.

Short-duration supplementation benefited only TGs, while long-duration supplementation was beneficial for TGs, TC, and LDL cholesterol. Furthermore, the effect of long-duration supplementation was greater than that of short-duration supplementation for all 3 of these risk factors. Neither short- nor long-duration supplementation had an effect on HDL cholesterol, SBP, or DBP.

## Discussion

This report first aimed to understand how dosage and duration of zinc supplementation impacted risk factors for T2D and CVD. Low-dose zinc supplementation benefitted 5 outcomes (FBG, HOMA-IR, TGs, TC, and LDL cholesterol) compared with placebo, while high-dose supplementation benefited 3 outcomes (HbA1c, HOMA-IR, and TGs). Additionally, low-dose supplementation showed a pattern of having larger ESs than high-dose supplementation for all 5 outcomes for which low-dose supplementation had a significant effect. Duration also impacted outcomes, with studies lasting <12 wk impacting 3 outcomes (FBG, HOMA-IR, and TGs), while studies lasting ≥12 wk had significant effects on 5 outcomes (FBG, HbA1c, TGs, TC, and LDL cholesterol). As observed in the dose analyses, there was a pattern of larger-magnitude ESs in long-duration studies compared with short-duration studies. No effects were observed for any dose or duration for HDL cholesterol, SBP, or DBP. Collectively, these findings suggest that, while high-dose and short-duration zinc interventions both benefit some risk factors for T2D and CVD, low doses of zinc and longer durations impact a greater number of these risk factors.

It is possible that low doses of zinc are more beneficial than high doses because zinc is known to display toxicity in excess quantities (20), which may partially explain the increase in risk reported in some studies when comparing those with the highest zinc intake with the lowest (49, 50). Low doses may also encourage higher fractional absorption, as high doses have been shown to lower absorption due to the saturable nature of the system (82). Initially, we hypothesized that the benefit of low-dose supplementation may be due to an association between dose and duration, with lower doses being effective because they were administered for longer durations. However, the Pearson correlation between dose and duration was only −0.27 and was not significant (*P* = 0.18), suggesting that dose and duration are not inherently related. Therefore, it seems that low doses of zinc, regardless of their duration, can be as or more beneficial for CVD and T2D risk factors than larger doses and may avoid potential negative side effects. While some previous studies have included duration of supplementation as a covariate in their analyses (83), to our knowledge, there are currently no published systematic reviews or meta-analyses that have stratified by duration of supplementation. However, due to the chronic nature of diseases like T2D and CVD, it is logical that longer-duration interventions would be needed to have a meaningful impact on their development.

### Biofortification and risk factors for T2D and CVD

The findings of this study establish a foundation for the second aim of this study: to discuss the potential for biofortification interventions to address risk factors for T2D and CVD. Before doing so, it is important to clarify that this study is not suggesting that zinc supplementation or biofortification could be used as the sole or even primary strategy for combating NCDs and their risk factors in the general population. Rather, we are interested in whether replacing traditional, nutrient-poor varieties of staple crops with zinc-biofortified varieties could complement other treatment and prevention strategies in a sustainable and accessible way to help reduce the risk of NCDs in low-resource populations that are at high risk of zinc deficiencies.

Biofortification of staple crops provides low doses of dietary zinc regularly and consistently over time. The results of the present meta-analyses suggest that this combination of low-dose, long-duration zinc intervention has the potential to benefit multiple risk factors for T2D and CVD related to both glycemic control and lipid metabolism. There are several key differences between supplemental zinc and biofortification interventions that could impact the effects observed from the additional zinc, including the dose and duration of zinc provided by a biofortification intervention as well as the form through which that zinc is provided.

The amount of zinc provided by biofortified crops is much smaller than that provided even by the “low-dose” arm of the present meta-analyses. Consumption of biofortified crops provides ∼5–9 mg additional Zn/d compared with consumption of a conventional crop (84). In contrast, studies in the “low-dose” intervention arm of the present study provided zinc in doses ranging from 9.2 to 25 mg/d. This difference clearly would impact the size of the effect that the additional zinc obtained from biofortified crops could have; however, the permanence of the intervention once adopted creates a duration that is far longer than supplementation interventions provide. Therefore, it is possible that, while effects may take longer to observe, the near-permanence of a zinc-biofortification intervention could partially counterbalance the lower dose and produce similar, although likely smaller, benefits for risk factors for T2D and CVD.

Additionally, the mode through which a zinc intervention is delivered may impact the effects that it has. The present meta-analyses included zinc-supplementation studies, in which zinc was provided as a supplement once or twice a day, usually without food. In contrast, biofortification by nature provides zinc as part of a staple food crop. Furthermore, that crop is consumed with most or all meals, providing multiple small doses of zinc throughout the day rather than 1 or 2 larger doses. While it is likely that the conditions produced by a zinc-biofortification intervention would have different absorptive properties than zinc provided as a supplement, it is not necessarily the case that these differences would prevent or alter the benefits observed from supplemental zinc in the present study. Indeed, 2 recent studies found that the fractional absorption of zinc from biofortified crops did not differ from that from fortified varieties (85, 86). They also found that the rate of extraction (80% vs 100%) had no effect on total absorbed zinc (85). However, the differences in fractional and total zinc absorption between zinc supplements and food-based interventions are less clear and could differ depending on the total elemental zinc content, degree of milling of the zinc-containing crop, type of compound, and whether the supplement was taken with or without food.

Despite these uncertainties, recent literature does suggest that zinc obtained from food-based sources (dietary zinc) can impact risk factors for NCDs. A recent meta-analysis by Fernández-Cao et al. (10) suggests that the effect of low-dose dietary zinc interventions may be similar to those observed in the present study. They reported that dietary zinc intakes that are near or slightly higher than the Institute of Medicine's Dietary Reference Intake (8 and 11 mg/d for females and males, respectively) (87) may be protective against the development of T2D when at least part of the population is not consuming zinc at the recommended level. This finding held even when intermediate quantiles were compared with the lowest quantile. Therefore, it is likely that even a modest increase in dietary zinc intake from the consumption of biofortified crops, which might move an individual from the lowest intake quantile to a middle quantile, could have a meaningful effect on their risk of developing T2D or other chronic diseases. However, future studies should examine whether the modality through which zinc is provided has an impact on the effects that it has on risk factors for NCDs.

### Physiological relation between zinc and NCDs

Given the known roles that zinc deficiency plays in the physiology of insulin homeostasis and lipid metabolism, it is not surprising that several studies have reported positive effects of zinc supplementation on the risk of T2D and CVD (16, 42–44). Wang et al. (88) found that zinc supplementation induces metallothionein expression and subsequently reduces diabetic vascular complications. Similarly, a recent meta-analysis by Khazdouz et al. (43) reported that zinc supplementation improved overall glycemic index, FBG, and HbA1c; improved TC, VLDL cholesterol, and TG concentrations; and had no effect on HOMA-IR, SBP, or DBP. The findings of the present analysis align with those of the Khazdouz et al. analysis in that FBG, HbA1c, TC, and TG concentrations were found to benefit from zinc supplementation and that blood pressure did not. However, the present study also found a beneficial effect of zinc supplementation on HOMA-IR and LDL cholesterol, while the Khazdouz et al. analysis did not. This discrepancy may be because the Khazdouz et al. study only included populations who had existing CVD, T2D, or obesity, whereas the current analysis included all populations regardless of their health status at baseline.

Patients with existing heath conditions like T2D have been shown to benefit more from supplemental or dietary zinc on risk factors for CVD and T2D than individuals who are not currently suffering from chronic diseases (11). However, that is not to say that those without existing conditions would not benefit from zinc interventions. Specifically, populations in low-resource, rural areas may still benefit greatly from zinc interventions. Diets of the rural poor in many developing countries lack sufficient zinc and contain high amounts of phytate that block zinc absorption, thus increasing their risk for zinc deficiency (89, 90). In addition, many of these low-resource regions are experiencing a double burden of malnutrition, further increasing their risk for NCDs (91). Existing interventions such as fortification, dietary diversification, or supplementation are often not sustainable or accessible for the rural poor in these regions (35). However, these populations may be among those who are most in need of zinc interventions.

For example, the meta-analysis by Fernández-Cao et al. (10) compared the T2D prevalence between the highest and lowest dietary zinc intake quantiles in urban versus rural areas and found that there was a significant protective effect of high dietary zinc intake in rural areas (OR: 0.59; 95% CI: 0.48, 0.73) while urban areas had a much smaller, nonsignificant effect (OR: 0.94; 95%: 0.86, 1.02) (10). This finding suggests that improving the dietary zinc intake of those in rural areas could be especially beneficial in addressing risk factors for NCDs. Biofortification is a preventative strategy that is meant to complement existing interventions specifically by reaching rural and underserved populations (36); therefore, it could serve as an excellent strategy for increasing dietary intake in rural populations. However, to date, no studies have been conducted examining the impact of biofortified crop consumption on risk factors for T2D or CVD—although there have been several efficacy trials examining the impact of zinc-biofortified crops on zinc status (37, 39) and health outcomes less relevant to NCDs.

A study by Sazawal et al. (37) reported that the consumption of zinc-biofortified wheat reduced the number of days with pneumonia, vomiting, or fever in Indian schoolchildren; however, the study did not look at chronic conditions. There is also a growing body of literature that indirectly addresses biofortification and NCD risk, such as the impact of low doses of zinc on plasma zinc, fatty acid metabolism, and DNA damage (92, 93)—research that has direct implications for dyslipidemia and insulin resistance.

### Future directions

Conducting this series of meta-analyses highlighted several critical gaps in the existing literature base surrounding zinc and risk of T2D and CVD.

First, there is an interesting discrepancy between the results of randomized zinc-supplementation trials or meta-analyses and those examining dietary intake in longitudinal cohorts. Several longitudinal cohort studies have examined the association between dietary zinc intake and risk of T2D. The Nurses’ Health Study reported an inverse relation between total and/or dietary zinc intake and the risk of T2D but found no evidence that zinc supplementation was related to T2D risk (94). However, they did report that supplementation may have a positive influence on the progression from prediabetes to T2D. Similarly, Fernández-Cao et al. (10) found a protective effect of dietary zinc intake (OR: 0.87; 95% CI: 0.78, 0.98; *P* = 0.003) when comparing the highest with the lowest dietary intake quantiles, but found no effect of supplemental zinc.

It is possible that supplemental zinc was not associated with risk of T2D in these longitudinal studies because supplemental zinc is not well represented among longitudinal cohort data. For example, only 3 studies reported supplement use in the Fernández-Cao et al. meta-analysis (10). This lack of data may be due to the difficulty of accurately measuring supplement use in large cohorts. Assessing supplement use (type, duration, and frequency) in cohort studies is difficult due to inconsistencies between the definitions of the term “supplement” and/or variations in what is considered “use” (daily, frequently, occasionally, etc.) (95). Supplement use reported in free-living conditions may also be less consistent than that received in a zinc-supplementation trial or may be combined with other micronutrients or supplements, which could explain why randomized supplementation trials have shown consistent benefits of zinc supplementation, while longitudinal cohort studies have not. Despite this discrepancy, randomized trials and meta-analyses of these trials suggest that increasing zinc (via supplementation) is an effective way to mitigate the risk of T2D and CVD. When viewed in combination with the results of the cohort studies, which show that higher dietary zinc consumption is associated with improved risk factors for T2D and CVD, these findings suggest that reducing zinc deficiency by any means could have meaningful impacts on risk factors for these conditions.

Second, existing zinc supplementation studies have generally used a wide range of supplement doses and durations. In the present study, doses ranged from 9.2 mg/d to as high as 75 mg/d with durations of 4 to 52 wk. While these dose and duration ranges are fairly wide, only 2 studies provided <20 mg/d and 3 studies had interventions lasting >12 wk. Therefore, our ability to understand the impact of very low doses of zinc provided over a longer duration of time is limited among randomized supplementation trials. We attempted to examine dose-by-duration effects in the meta-analyses; however, the sample size was insufficient when broken into the 4 dose-by-duration categories—with only 3 studies being low-dose, long-duration and 5 studies of low-dose, short-duration. This resulted in the majority of low-dose, long-duration and low-dose, short-duration studies having only 1 or 2 studies included for each outcome, severely limiting the power of these analyses. While it is reasonable that providing a longer-term zinc intervention would have a greater benefit than a shorter-duration intervention, it would be prudent for future research to examine the detailed effects of intervention duration in zinc interventions across a variety of populations. Additionally, as more research is published in this area and the number of studies in each dose-by-duration category increases, the meta-analyses in the present study should be re-evaluated as dose-by-duration analyses.

Third, a critical area that needs clarification is the impact of the baseline zinc status of an intervention population on the efficacy of zinc interventions on chronic disease risk factors. Only 2 of the 27 studies in the present analyses explicitly stated that their participants were below the serum zinc concentration considered normal by each study's authors (68, 75). Due to the challenges in assessing zinc status at the individual level (96), it can be difficult to determine whether participants in zinc-intervention trials are at risk for or are currently affected by insufficient zinc intake. Furthermore, several studies have reported that they observed significant changes in morbidity from zinc supplementation, even when there were no significant changes in serum zinc concentrations (93, 97). Future studies that are specifically interested in understanding how biofortification may affect NCDs may want to consider making risk factors for T2D or CVD their primary outcomes, rather than serum zinc concentration, which may not be a sensitive or appropriate outcome measure for these conditions or for interventions with low amounts of dietary (nonsupplementation) zinc.

Finally, the present study included publications involving all populations—with or without existing health conditions. Currently, most biofortification interventions focus on populations at risk of zinc deficiency but do not currently target populations with other conditions such as T2D, CVD, or metabolic diseases. Given the known relations between weight, inflammation, and zinc metabolism (16, 20, 46), it would be interesting for future studies to examine the differences in the response to consuming zinc-biofortified products and the development or progression of these conditions in populations with overweight or obesity and/or specific medical conditions.

### Limitations

This study had several limitations. First, there are currently no studies that have directly examined the impact of biofortified crop or fortified food consumption on the incidence of, or risk factors for, T2D and CVD. Therefore, supplemental zinc studies were used as a starting point for understanding the impact that low-dose or long-duration zinc interventions could have on risk factors for these conditions. In addition to the differences in dose, duration, and mode of delivery between supplemental and biofortified zinc discussed previously, one limitation to this approach is that supplementation trials are much more controlled than the real-world settings in which biofortified crops would be consumed. Any of these differences could potentially alter the impact of low-dose and long-duration zinc on risk factors for T2D and CVD. However, supplementation trials were selected for this review because we were interested in understanding the potential effect that low-dose or long-duration zinc interventions could have on these conditions (similar to an efficacy study) rather than the impact it would have in real-world conditions (similar to an effectiveness study). This study was conducted to generate hypotheses; therefore, the basic question of whether lower doses of zinc over long periods of time needed to be answered before more generalizable questions could be asked.

Second, several of the meta-analyses included only a small number of studies. A small sample size may limit the power to detect potential confounding factors or sources of heterogeneity. We attempted to address this issue by using random-effects meta-analyses.

### Conclusions

This analysis was conducted to first examine the effects of dose and duration on the impact of zinc supplementation on risk factors for T2D and CVD and to evaluate the potential for zinc-biofortification interventions to impact these risk factors. To do this, we conducted a series of meta-analyses of zinc-supplementation trials to understand the role of dose and duration of supplementation on various risk factors for T2D and CVD. The analyses showed that lower doses and longer durations both affected a greater number of risk factors than interventions providing higher doses or using shorter durations. Collectively, the results of the present study and others (10, 16, 42, 92, 93, 97) support the idea that the modest increase in absorbable dietary zinc intake resulting from the consumption of biofortified crops could elicit meaningful impacts on lipid metabolism, DNA repair, redox balance, and inflammation that could ultimately benefit risk factors for T2D and CVD.

It is critically important that strategies be developed to address zinc deficiency in all populations, including those who are not currently benefiting from traditional zinc intervention methodologies like supplementation or fortification. Biofortification has been shown to improve zinc status in at-risk populations and to have impacts on some measures of morbidity (37, 39, 92, 93). However, to fully understand the impact that zinc-biofortified crops could have on the double burden of disease, future long-term randomized trials or cohort studies should be conducted that specifically examine risk factors for T2D and CVD as primary outcomes.

## Supplementary Material

nmaa087_Supplemental_File

## Notes

The authors reported no funding received for this study.

Author disclosures: The authors report no conflicts of interest.

Supplemental Table 1 and Supplemental Figures 1–3 are available from the “Supplementary data” link in the online posting of the article and from the same link in the online table of contents at https://academic.oup.com/advances.

Abbreviations used: CVD, cardiovascular disease; DBP, diastolic blood pressure; ES, effect size; FBG, fasting blood glucose; HbA1c, glycated hemoglobin; NCD, noncommunicable disease; SBP, systolic blood pressure; TC, total cholesterol; TG, triglyceride; T2D, type 2 diabetes.
