# Supplementary material for: Effects of Dose and Duration of Zinc Interventions on Risk Factors for Type 2 Diabetes and Cardiovascular Disease: A Systematic Review and Meta-Analysis
Source: Adv Nutr. 2020 Jul 28;12(1):141–60. doi: 10.1093/advances/nmaa087 (PMC7850144; doi:10.1093/advances/nmaa087)
Supplement: nmaa087_Supplemental_File [file nmaa087_supplemental_file.pdf]

Title: The effects of dose and duration of zinc interventions on risk factors for type 2 diabetes mellitus and cardiovascular disease: A systematic review and meta-analysis

Pompano and Boy – Online Supplementary Material

Supplemental Table 1: PICOS criteria followed in the review
Abbreviations: PICOS – Population, Intervention, Comparison, Outcome, Study Design

**Parameters**	**Inclusion Criteria**	**Exclusion Criteria**
Participants	Human subjects of any age, sex, or BMI	
Interventions	Provided zinc alone in any form in any dosage between 1 and 100 mg/day	Provided zinc in combination with any other micronutrient, compound, or drug
Comparisons	Provided any control or placebo	Provided control/placebo in combination with any other micronutrient, compound, or drug<br><br>Did not have a control or placebo group
Outcomes	Evaluated at least one of the following outcomes: fasting blood glucose, glycated hemoglobin, insulin resistance, triglycerides, total cholesterol, LDL-cholesterol, HDL-cholesterol, systolic blood pressure, or diastolic blood pressure	
Study Design	Controlled Trial providing post-intervention data for at least one outcome of interest	Studies not providing post-intervention data

Supplemental Figure 1: Funnel plots for publication bias

Fasting Blood Glucose – High Dose (*n* = 15)

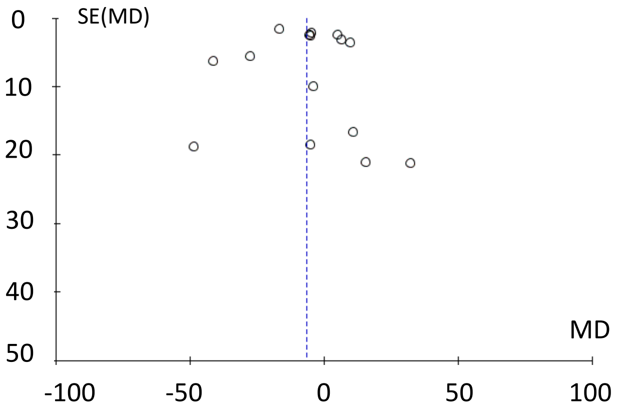

Fasting Blood Glucose – Short Duration (*n* = 12)

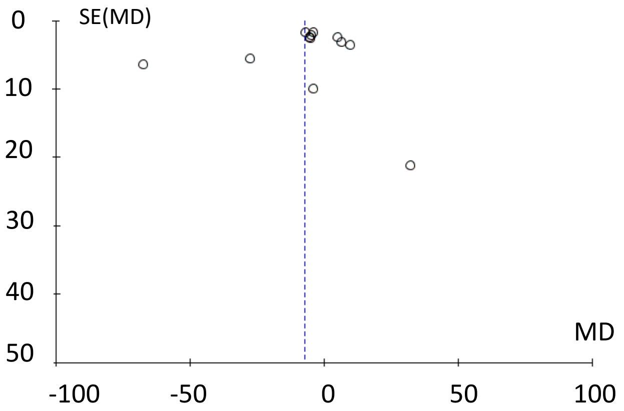

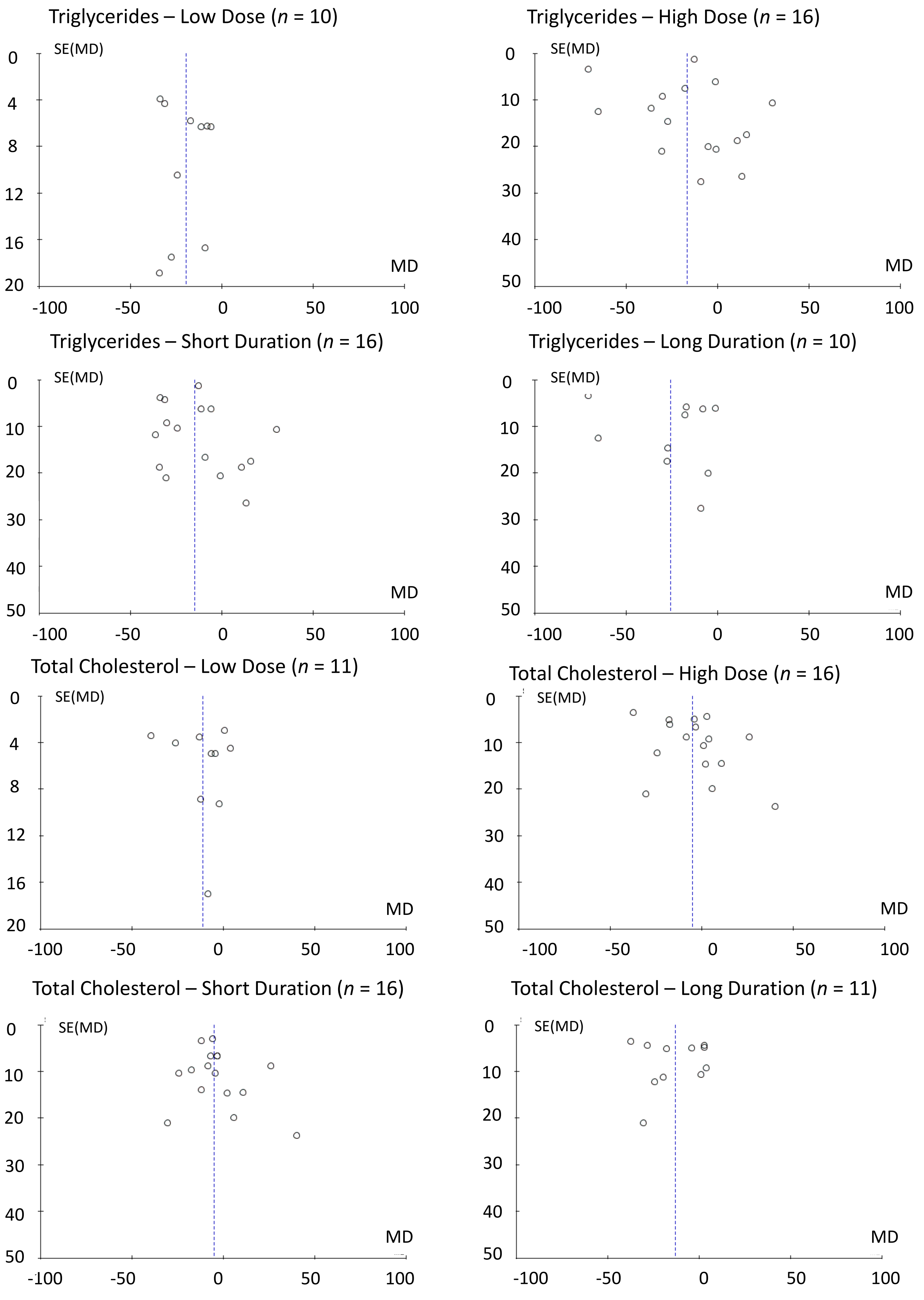

Triglycerides – Low Dose (n = 10)
SE(MD)
0
4
8
12
16
20
MD
-100
-50
0
50
100
Triglycerides – High Dose (n = 16)
SE(MD)
0
10
20
30
40
50
MD
-100
-50
0
50
100
Triglycerides – Short Duration (n = 16)
SE(MD)
0
10
20
30
40
50
MD
-100
-50
0
50
100
Triglycerides – Long Duration (n = 10)
SE(MD)
0
10
20
30
40
50
MD
-100
-50
0
50
100
Total Cholesterol – Low Dose (n = 11)
SE(MD)
0
4
8
12
16
20
MD
-100
-50
0
50
100
Total Cholesterol – High Dose (n = 16)
SE(MD)
0
10
20
30
40
50
MD
-100
-50
0
50
100
Total Cholesterol – Short Duration (n = 16)
SE(MD)
0
10
20
30
40
50
MD
-100
-50
0
50
100
Total Cholesterol – Long Duration (n = 11)
SE(MD)
0
10
20
30
40
50
MD
-100
-50
0
50
100

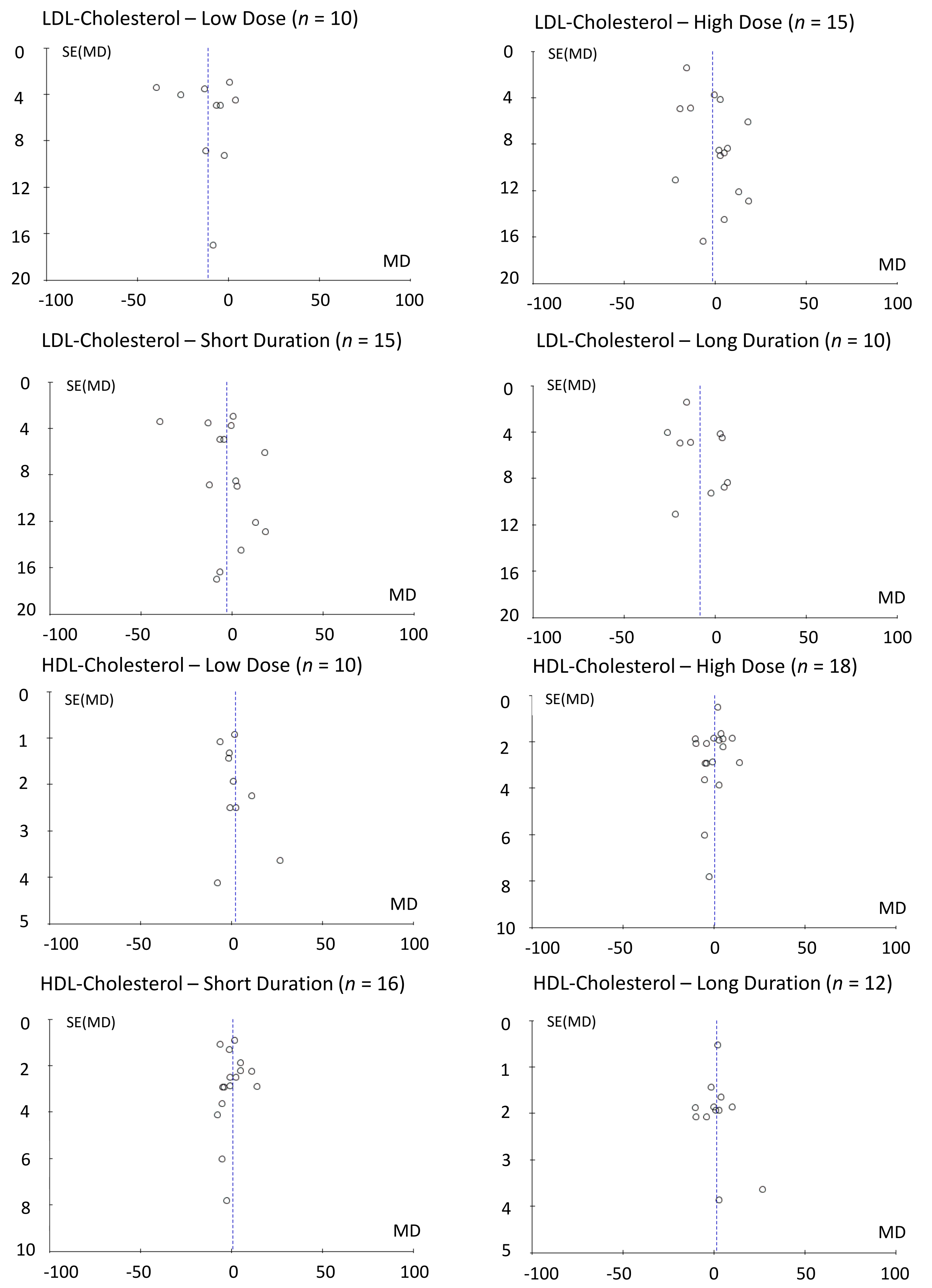

LDL-Cholesterol – Low Dose (n = 10)
LDL-Cholesterol – High Dose (n = 15)
LDL-Cholesterol – Short Duration (n = 15)
LDL-Cholesterol – Long Duration (n = 10)
HDL-Cholesterol – Low Dose (n = 10)
HDL-Cholesterol – High Dose (n = 18)
HDL-Cholesterol – Short Duration (n = 16)
HDL-Cholesterol – Long Duration (n = 12)
SE(MD)
MD

Supplemental Figure 2: Meta-analyses of the mean difference between zinc and placebo on systolic blood pressure, by dose and duration[1]

A

Study or Subgroup	Zn Mean	Zn SD	Zn Total	Control Mean	Control SD	Control Total	Weight	Mean Difference IV, Random, 95% CI
Hashemipour et al. (58) - Placebo first	93.21	10.12	30	96.88	10.3	30	28.6%	-3.67 [-8.84, 1.50]
Hashemipour et al. (58) - Zinc first	96.88	10.3	30	94.21	10.12	30	28.6%	2.67 [-2.50, 7.84]
Ranasinghe et al. (81)	123.3	13.2	73	124.6	9.3	65	42.8%	-1.30 [-5.08, 2.48]
**Total (95% CI)**			**133**			**125**	**100.0%**	**-0.84 [-4.11, 2.43]**

Heterogeneity: Tau² = 2.78; Chi² = 2.98, df = 2 (P = 0.23); I² = 33%
Test for overall effect: Z = 0.51 (P = 0.61)

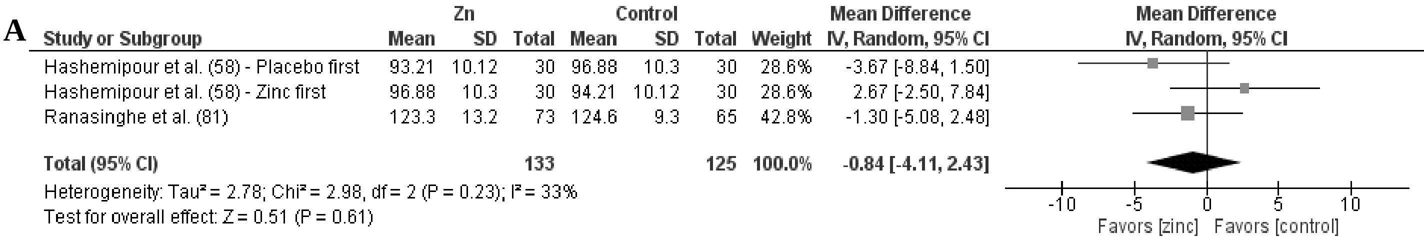

B

Study or Subgroup	Zn Mean	Zn SD	Zn Total	Control Mean	Control SD	Control Total	Weight	Mean Difference IV, Random, 95% CI
Kim and Lee (67)	117.98	9.51	20	117.1	12.93	20	27.0%	0.88 [-6.15, 7.91]
Parham et al. (59) - Placebo first	123	10	18	125.5	14	21	23.4%	-2.50 [-10.06, 5.06]
Parham et al. (59) - Zinc first	119	9.5	21	118	7	18	49.6%	1.00 [-4.19, 6.19]
**Total (95% CI)**			**59**			**59**	**100.0%**	**0.15 [-3.51, 3.81]**

Heterogeneity: Tau² = 0.00; Chi² = 0.62, df = 2 (P = 0.73); I² = 0%
Test for overall effect: Z = 0.08 (P = 0.94)

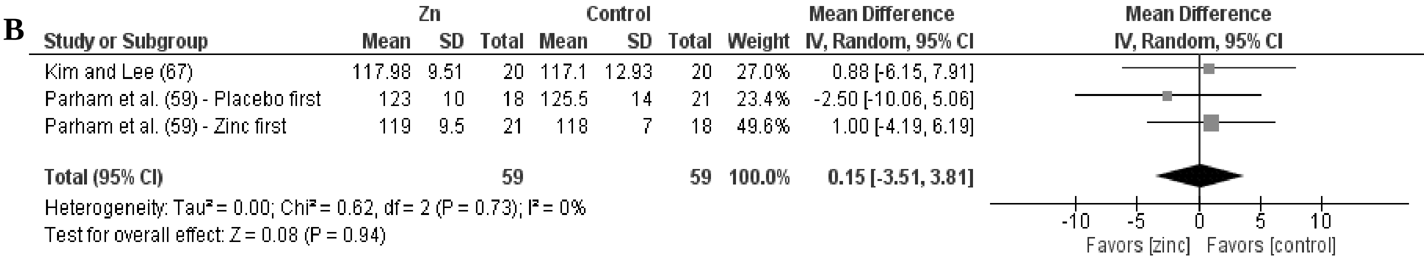

C

Study or Subgroup	Zn Mean	Zn SD	Zn Total	Control Mean	Control SD	Control Total	Weight	Mean Difference IV, Random, 95% CI
Hashemipour et al. (58) - Placebo first	93.21	10.12	30	96.88	10.3	30	37.7%	-3.67 [-8.84, 1.50]
Hashemipour et al. (58) - Zinc first	96.88	10.3	30	94.21	10.12	30	37.7%	2.67 [-2.50, 7.84]
Kim and Lee (67)	117.98	9.51	20	117.1	12.93	20	24.7%	0.88 [-6.15, 7.91]
**Total (95% CI)**			**80**			**80**	**100.0%**	**-0.16 [-4.19, 3.87]**

Heterogeneity: Tau² = 4.28; Chi² = 3.01, df = 2 (P = 0.22); I² = 34%
Test for overall effect: Z = 0.08 (P = 0.94)

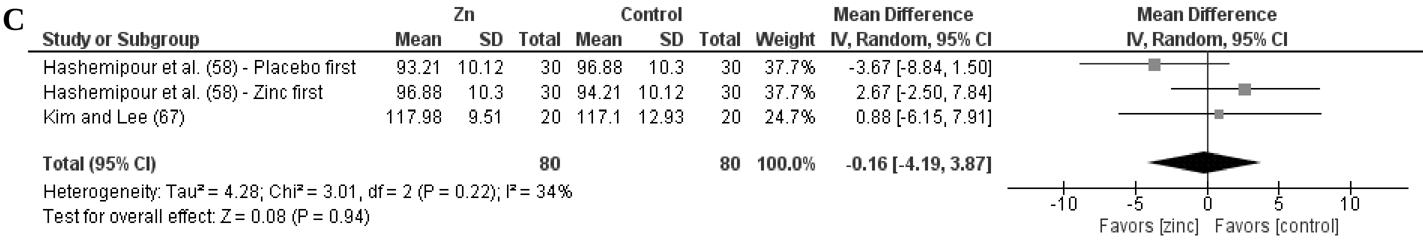

D

Study or Subgroup	Zn Mean	Zn SD	Zn Total	Control Mean	Control SD	Control Total	Weight	Mean Difference IV, Random, 95% CI
Parham et al. (59) - Placebo first	123	10	18	125.5	14	21	14.0%	-2.50 [-10.06, 5.06]
Parham et al. (59) - Zinc first	119	9.5	21	118	7	18	29.8%	1.00 [-4.19, 6.19]
Ranasinghe et al. (81)	123.3	13.2	73	124.6	9.3	65	56.2%	-1.30 [-5.08, 2.48]
**Total (95% CI)**			**112**			**104**	**100.0%**	**-0.78 [-3.62, 2.05]**

Heterogeneity: Tau² = 0.00; Chi² = 0.72, df = 2 (P = 0.70); I² = 0%
Test for overall effect: Z = 0.54 (P = 0.59)

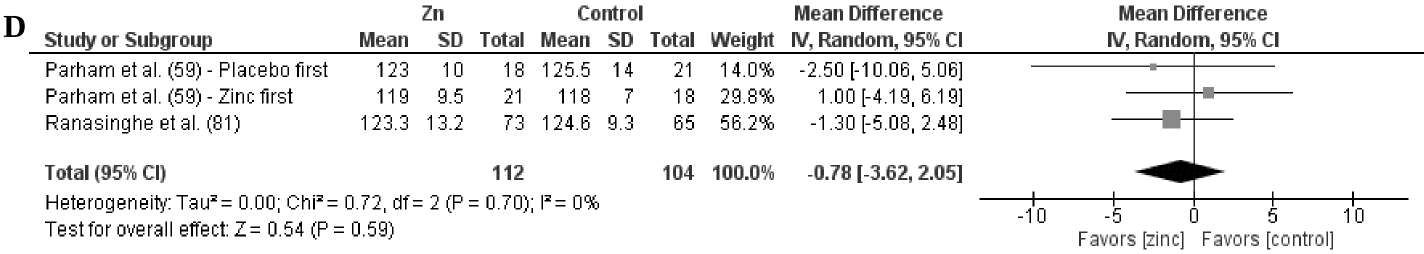

[1]Panel A: Studies providing low-dose supplementation, defined as <25mg elemental zinc per day; Panel B: Studies providing high-dose supplementation, defined as ≥25mg elemental zinc per day; Panel C: Studies providing short-duration supplementation, defined as <12 weeks; Panel D: Studies providing long-duration supplementation, defined as ≥12 weeks

Supplemental Figure 3: Meta-analyses of the mean difference between zinc and placebo on diastolic blood pressure, by dose and duration[1]

A

Study or Subgroup	Zn Mean	Zn SD	Zn Total	Control Mean	Control SD	Control Total	Weight	Mean Difference IV, Random, 95% CI
Hashemipour et al. (58) - Placebo first	50	10.38	30	54.17	6.54	30	24.9%	-4.17 [-8.56, 0.22]
Hashemipour et al. (58) - Zinc first	54.17	6.54	30	54	10.38	30	24.9%	0.17 [-4.22, 4.56]
Ranasinghe et al. (81)	77.3	11.4	73	77.8	4.8	65	50.2%	-0.50 [-3.36, 2.36]
**Total (95% CI)**			**133**			**125**	**100.0%**	**-1.25 [-3.58, 1.09]**

Heterogeneity: Tau² = 0.69; Chi² = 2.36, df = 2 (P = 0.31); I² = 15%
Test for overall effect: Z = 1.05 (P = 0.30)

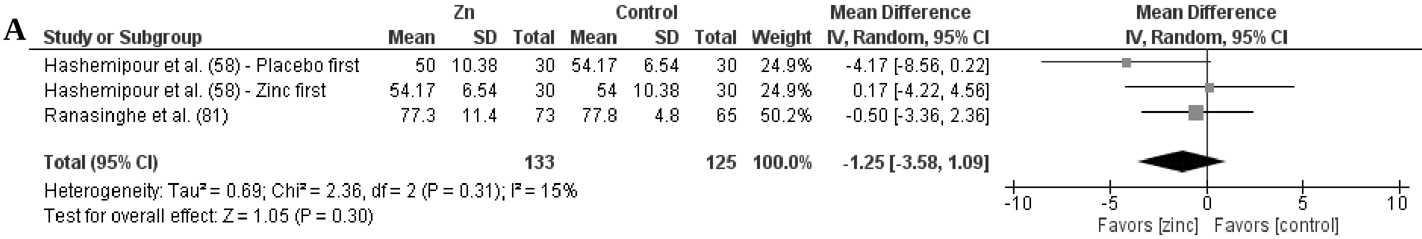

B

Study or Subgroup	Zn Mean	Zn SD	Zn Total	Control Mean	Control SD	Control Total	Weight	Mean Difference IV, Random, 95% CI
Kim and Lee (67)	78.23	8.11	20	76.7	8.08	20	26.3%	1.53 [-3.49, 6.55]
Parham et al. (59) - Placebo first	75	6	18	77	16	21	12.1%	-2.00 [-9.38, 5.38]
Parham et al. (59) - Zinc first	75.5	6.1	21	73	4.3	18	61.6%	2.50 [-0.78, 5.78]
**Total (95% CI)**			**59**			**59**	**100.0%**	**1.70 [-0.87, 4.27]**

Heterogeneity: Tau² = 0.00; Chi² = 1.20, df = 2 (P = 0.55); I² = 0%
Test for overall effect: Z = 1.29 (P = 0.20)

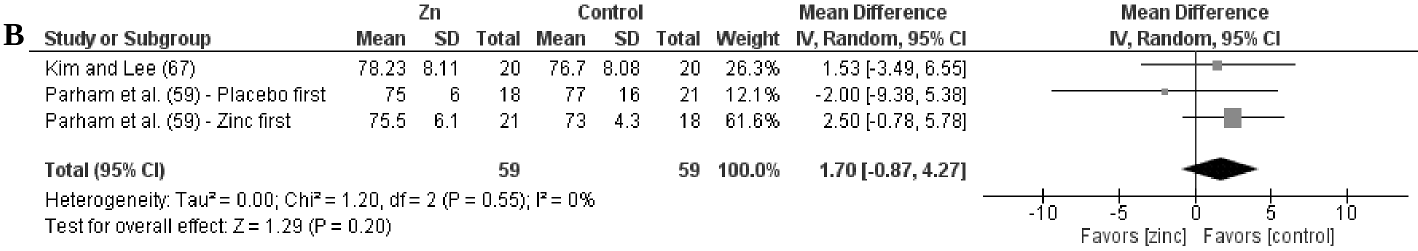

C

Study or Subgroup	Zn Mean	Zn SD	Zn Total	Control Mean	Control SD	Control Total	Weight	Mean Difference IV, Random, 95% CI
Hashemipour et al. (58) - Placebo first	50	10.38	30	54.17	6.54	30	35.1%	-4.17 [-8.56, 0.22]
Hashemipour et al. (58) - Zinc first	54.17	6.54	30	54	10.38	30	35.1%	0.17 [-4.22, 4.56]
Kim and Lee (67)	78.23	8.11	20	76.7	8.08	20	29.7%	1.53 [-3.49, 6.55]
**Total (95% CI)**			**80**			**80**	**100.0%**	**-0.95 [-4.33, 2.43]**

Heterogeneity: Tau² = 3.43; Chi² = 3.25, df = 2 (P = 0.20); I² = 39%
Test for overall effect: Z = 0.55 (P = 0.58)

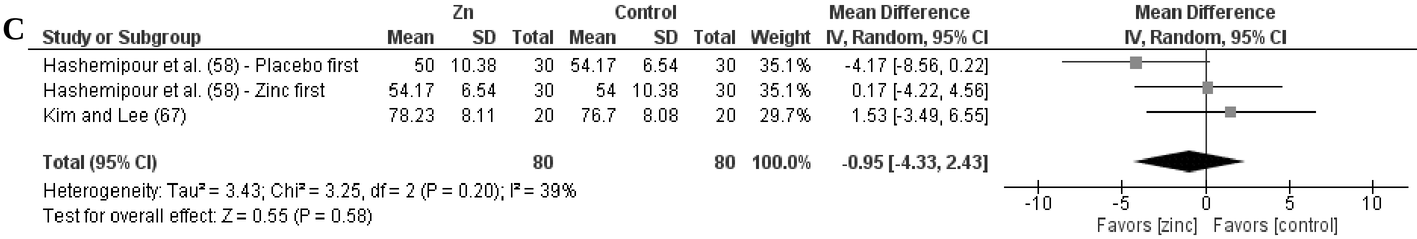

D

Study or Subgroup	Zn Mean	Zn SD	Zn Total	Control Mean	Control SD	Control Total	Weight	Mean Difference IV, Random, 95% CI
Parham et al. (59) - Placebo first	75	6	18	77	16	21	9.4%	-2.00 [-9.38, 5.38]
Parham et al. (59) - Zinc first	75.5	6.1	21	73	4.3	18	40.5%	2.50 [-0.78, 5.78]
Ranasinghe et al. (81)	77.3	11.4	73	77.8	4.8	65	50.1%	-0.50 [-3.36, 2.36]
**Total (95% CI)**			**112**			**104**	**100.0%**	**0.57 [-1.75, 2.89]**

Heterogeneity: Tau² = 0.66; Chi² = 2.33, df = 2 (P = 0.31); I² = 14%
Test for overall effect: Z = 0.48 (P = 0.63)

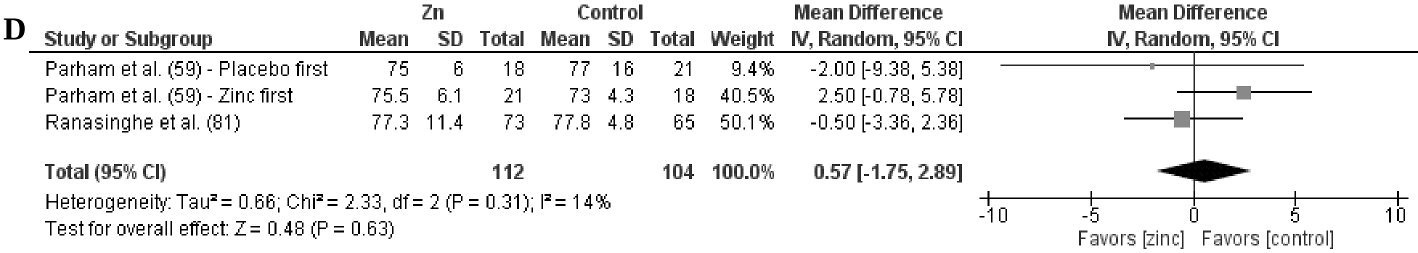

[1]Panel A: Studies providing low-dose supplementation, defined as <25mg elemental zinc per day; Panel B: Studies providing high-dose supplementation, defined as ≥25mg elemental zinc per day; Panel C: Studies providing short-duration supplementation, defined as <12 weeks; Panel D: Studies providing long-duration supplementation, defined as ≥12 weeks
